# Severe ciliopathy-related phenotypes in mice with dysregulation of tubulin polyglutamylation

**DOI:** 10.1186/2046-2530-1-S1-P90

**Published:** 2012-11-16

**Authors:** K Ikegami, A Konno, S Hattori, C Matsuda, M Setou

**Affiliations:** 1 Hamamatsu University School of Medicine, Japan

## 

Tubulin, a main component of ciliary and flagellar axonemes, undergoes highly unique post-translational modifications, polyglutamylation and polyglycylation. Recent years, evidence accumulates that dysregulations of these two modifications lead to severe ciliary defects in a variety of model organisms, such as *Chlamydomonas*, *Tetrahymena*, *C. elegans*, *Drosophila*, and zebrafish. Previously, we have for the first time revealed that a reduction of tubulin polyglutamylation causes ciliopathy-related defects including severe respiratory problems, such as paranasal sinusitis and repetitive coughing or sneezing, and male infertility by means of a knockout mouse of a glutamate ligase (*TTLL1KO*) [Ikegami et al. 2010 PNAS]. Despite the clear ciliopathy-related defects by the loss of polyglutamylation-performing enzyme, it is still veiled whether over-polyglutamylation leads to ciliopathy-related phenotypes in mice. To address the question, we examined the retina of a spontaneous mutant of a glutamate-removing enzyme (*pcd*) mouse that displays late-onset retinal photoreceptor degeneration. The *pcd* mouse showed stronger polyglutamylation signals in the retinal cone and rod layer compared to wild-type animal. To test if the hyper-polyglutamylation leads to retinal degeneration, we generated a double mutant of *pcd* and *TTLL1KO*. The hyper-polyglutamylation observed in the cone and rod layer of *pcd* mice was neutralized in that of *pcd/TTLL1KO* double mutant. The retinal photoreceptor degeneration in *pcd* was almost completely rescued in the *pcd/TTLL1KO* double mutant. These results suggest that hyper-polyglutamylation underlies retinal photoreceptor degeneration. We would emphasize, in the conference, the importance of keeping narrow range of polyglutamylation level to maintain ciliary function.

